# Bio-Inspired Curved-Elliptical Lattice Structures for Enhanced Mechanical Performance and Deformation Stability

**DOI:** 10.3390/ma17174191

**Published:** 2024-08-24

**Authors:** Zhengmiao Guo, Fan Yang, Lingbo Li, Jiacheng Wu

**Affiliations:** 1 School of Aerospace Engineering and Applied Mechanics, Tongji University, Shanghai 200092, China; 2230876@tongji.edu.cn (Z.G.); 2210148@tongji.edu.cn (L.L.); 2311358@tongji.edu.cn (J.W.); 2 Key Laboratory of AI-Aided Airworthiness of Civil Aircraft Structures, Civil Aviation Administration of China, Tongji University, Shanghai 200092, China; 3 State Key Laboratory of Structural Analysis, Optimization and CAE Software for Industrial Equipment, Dalian University of Technology, Dalian 116024, China

**Keywords:** lattice structure, energy absorption, stiffness, delocalized deformation

## Abstract

Lattice structures, characterized by their lightweight nature, high specific mechanical properties, and high design flexibility, have found widespread applications in fields such as aerospace and automotive engineering. However, the lightweight design of lattice structures often presents a trade-off between strength and stiffness. To tackle this issue, a bio-inspired curved-elliptical (BCE) lattice is proposed to enhance the mechanical performance and deformation stability of three-dimensional lattice structures. BCE lattice specimens with different parameters were fabricated using selective laser melting (SLM) technology, followed by quasi-static compression tests. Finite element (FE) numerical simulations were also carried out for validation. The results demonstrate that the proposed BCE lattice structures exhibit stronger mechanical performance and more stable deformation modes that can be adjusted through parameter tuning. Specifically, by adjusting the design parameters, the BCE lattice structure can exhibit a bending-dominated delocalized deformation mode, avoiding catastrophic collapse during deformation. The specific energy absorption (SEA) can reach 24.6 J/g at a relative density of only 8%, with enhancements of 48.5% and 297.6% compared with the traditional energy-absorbing lattices Octet and body-center cubic (BCC), respectively. Moreover, the crushing force efficiency (CFE) of the BCE lattice structure surpasses those of Octet and BCC by 34.9% and 15.8%, respectively. Through a parametric study of the influence of the number of peaks *N* and the curve amplitude *A* on the compression performance of the BCE lattice structure, the compression deformation mechanism is further analyzed. The results indicate that the curve amplitude *A* and the number of peaks *N* have significant impacts on the deformation mode of the BCE lattice. By adjusting the parameters *N* and *A*, a structure with a combination of high energy absorption, high stiffness, and strong fracture resistance can be obtained, integrating the advantages of tensile-dominated and bending-dominated lattice structures.

## 1. Introduction

Lattice structures are periodic structures composed of a number of identical cells. Compared with traditional monolithic materials, lattice structures have a lower density, conducive to the lightweight design. In contrast to metal foams, lattice structures exhibit higher stiffness and strength [1]. Lattice structures possess superior mechanical properties such as high specific strength [2], high specific stiffness [3], and a strong energy absorption capability [4], among others [5,6,7], making them widely used in the aerospace [8,9,10], biomedical [11], and defense industries [12,13,14,15].

Over the past few decades, researchers have paid extensive attention to the mechanical performance of three-dimensional lattice structures, including truss-based lattice structures [16,17], plate-based lattice structures [18,19], and shell-based lattice structures [20,21]. Novel designs have been proposed to further optimize their mechanical properties, including biomimetic lattice structures [22], topologically graded lattice structures [23], and hierarchical lattice structures [24]. Notably, in recent years, the biomimetic design strategy has gained considerable attention in obtaining lightweight structures with excellent energy absorption capabilities in engineering fields. Lattice structures designed using biomimicry methods have demonstrated advantages over traditional structures. Plessis et al. [25] designed lattice structures that offer high strength and energy absorption. Han et al. [26] mimicked the lattice structures of two natural solids and introduced a new type of auxiliary material with ultra-high strength and ductility. In recent years, researchers have conducted biomimetic studies on deep-sea glass sponges [27,28,29,30], producing several biomimetic lattice structures. These advanced design strategies provide promising prospects for obtaining high-performance structural metamaterials. With the advancements in additive manufacturing technology [31,32], the possibility of designing novel lightweight lattice structures with superior performance based on biomimetic approaches has been greatly increased.

For three-dimensional truss lattice structures, replacing traditional straight bars with smooth curved beams has become an important strategy. Alomar et al. [33] developed a novel lattice structure based on circular elements. Studies have shown that this structure exhibits stretching-dominated behavior and performs well in shock absorption applications via layer-by-layer fracture. Li et al. [34] paid attention to the high concentration of stress at pillar nodes during the compression processes. They proposed three node reinforcement strategies to reduce the stress concentration, including adding fillets at the nodes, using curved beams, and altering node angles. Results indicate that the deformation mode of the optimized structure shifts from shear failure to layer-by-layer collapse, leading to a significant improvement in mechanical performance. Wang et al. [35] drew inspiration from the hierarchical structure of biological materials and proposed a novel hierarchical ring-shaped configuration for lattice structures. The designed multilevel circular-hole lattice structure exhibits more uniform stress distribution, with higher energy absorption capacity and enhanced mechanical performance. Zhu et al. [36] introduced a new lattice structure called the cosine function cell-based (CFCB) lattice, which exhibits an energy absorption of more than twice that of the conventional BCC lattice structure. While significant progress has been made in the study of curved beam lattice structures, most of the existing structures exhibit shear bands or layer-by-layer collapse during compression. Previous research focuses on the load-bearing capacity to address the stiffness drop due to the introduction of the initial curvature, while overlooking the stability of the compression process. The deformation instability featured by the deformation localization can lead to the loss of load-bearing capacity [37], resulting in significant oscillations in the stress response of the structure during compression. Therefore, it is important to design lattice structures with stable and controllable delocalized deformation. 

In this study, a bio-inspired lattice structure with elliptically curved beams is designed to combine the high load-bearing capability and strong deformation stability. For this purpose, we resort to the natural structures found in animals and plants to mimic the structural features that are conducive to high energy absorption and stable controllable deformation patterns. For example, the human spine consists of 33 different vertebrae interconnected by ligaments, small joints, and intervertebral discs, forming four physiological curves. It indicates that the curves can increase the flexibility and shock resistance of the spine [38]. Researchers also investigated the compression behavior of layered foam column structures inspired by sponge gourds. The biomimetic structures showed a significant increase in SEA [39]. In this paper, inspired by both zoic spines and botanic sponge gourds, we designed the BCE lattice structures in order to incorporate the advantages of both features. We used 316L stainless steel as the base material and employed SLM technology to fabricate the BCE specimens with different parameters. The specimens were subjected to quasi-static compression tests, and finite element models were established for simulating the compression process. The proposed BCE lattice structures were compared with traditional structures in terms of specific stiffness, SEA, crushing force efficiency, and deformation pattern. Furthermore, a parametric analysis was performed to investigate the effects of the number and amplitude of curved waves in both horizontal and vertical directions on the deformation mode and compression performance of the BCE lattice structure. 

## 2. Methods

### 2.1. Design Strategy for BCE Lattice Structures

**Figure 1:**
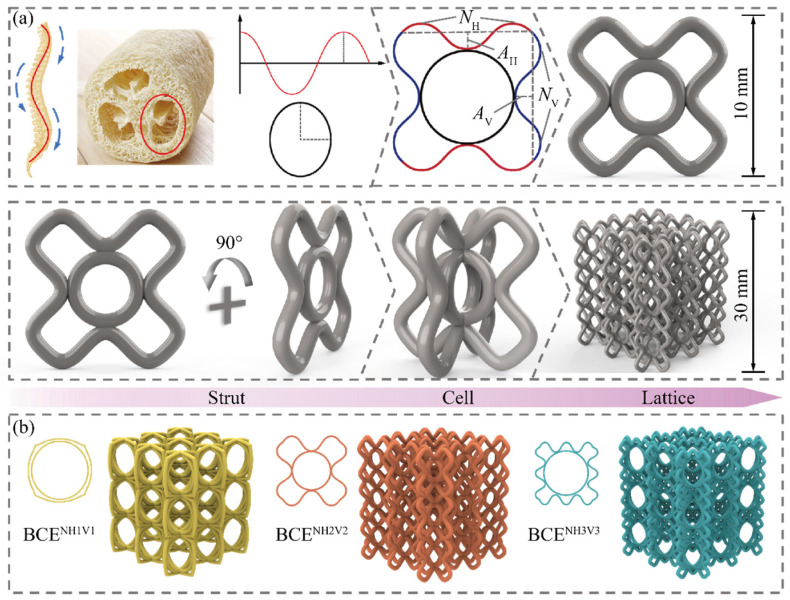
Geometry of the BCE lattice structure: (**a**) design strategy; (**b**) three examples of 3D BCE lattices.

Figure 1a presents the design strategy of the BCE lattice structure, where the curve and ellipse features are inspired by the zoic spines and the botanic sponge gourds, respectively. The cosine-shaped curves along the horizontal (*y_H_*) and vertical (*y_V_*) directions are displayed in red and blue, respectively. They are given by the following formulas which were also adopted in the literature [40]:

(1) $$y_{i}={\left(-1\right)}^{N_{i}+1}A_{i}\cos\left[\frac{\pi x_{i}(2N_{i}-1)}{2(L_{0}/2-A_{i})}\right]+\frac{L_{0}}{2}-A_{i}$$

In Equation (1), the subscripts *i* = H and *i* = V correspond to the horizontal and vertical directions, respectively. For example, *A*_H_ represents the amplitude of the cosine-shaped curve along the horizontal direction, while *A*_V_ represents the amplitude of the cosine-shaped curve along the vertical direction. *N_i_* denotes the amount of waviness of the curved beam. *L*_0_ denotes the side length of the unit cell. The shape and dimensions of the ellipse are fully determined by *A_i_* and *N_i_*, and the dimensions of the 2D unit cell containing the curves and ellipses are kept at 10 × 10 mm. The 2D unit cell is then combined with itself and rotated by 90° to form the 3D unit cell with dimensions of 10 × 10 × 10 mm. The lattice specimen containing a 3 × 3 × 3 array of unit cells has dimensions of 30 × 30 × 30 mm. Figure 1b depicts three examples of the BCE lattices, denoted as BCE^NH1V1^, BCE^NH2V2^, and BCE^NH3V3^, where BCE^NH1V1^ denotes the BCE lattice with *N_H_* = 3 and *N_V_* = 3, and so forth. 

### 2.2. Experimental Tests and Numerical Modeling 

**Figure 2:**
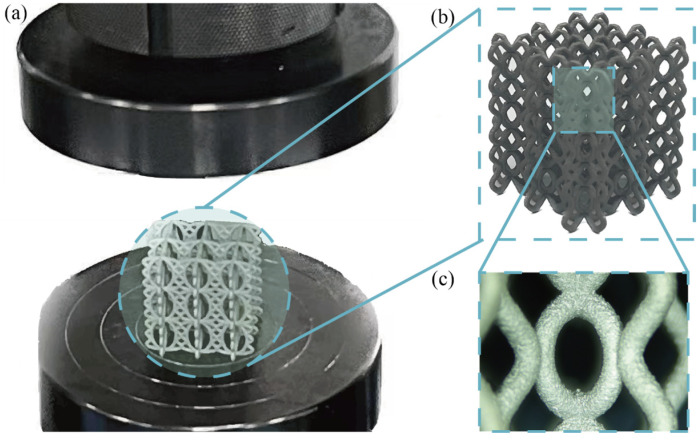
Quasi-static compression test and specimens: (**a**) test platform; (**b**) the BCE^NH2V2^ lattice specimen; (**c**) microscopic view of the specimen components.

To investigate the compression performance of BCE, a series of specimens with different geometric parameters were established, as shown by the three examples in Figure 1b. The specimen BCE^NH2V2^ is chosen as the benchmark case. The strut diameter of BCE^NH2V2^ is set as 1 mm, rendering a relative density of approximately 8%. In order to study the effect of the amount of waviness of the curved beams on the deformation mode and compression performance, nine BCE specimens were created with the amount of waviness along the horizontal and vertical directions varied as 1, 2, and 3. The same relative density is maintained by adjusting the strut diameter. The specimens were then manufactured using SLM additive manufacturing technology. SLM offers advantages such as the ability to achieve complex geometries, adaptability to various metal substrates, and high production efficiency. The base material of the 3D-printed specimens is 316L stainless steel. Table 1 gives the basic parameters of the SLM. During the fabrication process, the layer thickness ranged from 20 to 90 μm, the beam focusing diameter ranged from 80 to 115 μm, and the metal powder particle size ranged from 10 to 45 μm. The printing accuracy is maintained with a deviation of 0.05 mm per 100 mm. After printing, the specimens undergo a thermal treatment at approximately 1000 °C for one hour to reduce the residual stresses introduced during the printing process. Finally, a surface sandblasting treatment is applied to improve the surface finish, making the surface roughness of the sandblasted specimens approximately Ra10. Figure 2a,b show an example of the SLM-fabricated BCE specimens, BCE^NH2V1-3^, while Figure 2c displays a magnified view using an electron microscope (Dino-Lite, JIANGSU, CHINA). The quasi-static compression tests were conducted using the LE3000 universal testing machine (LISHI INSTRUMENT, SHANGHAI, CHINA) with a maximum capacity of 50 kN. To meet the quasi-static conditions, the compression speed was set to 5 mm/min.

**Figure 3:**
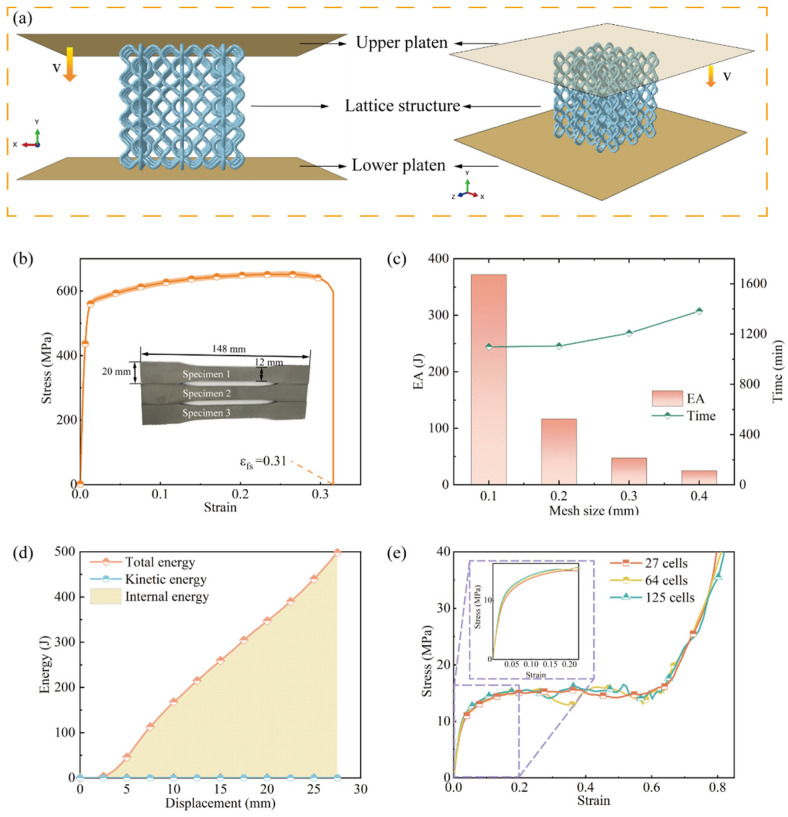
FE numerical model: (**a**) loading and boundary conditions; (**b**) true stress–strain curves of the base material (316L steel); (**c**) mesh convergence analysis; (**d**) energy comparison during the compression; (**e**) the boundary effect.

The quasi-static compression process was numerically simulated using the commercial FE software Abaqus (2020, Dassault Systèmes, French). Figure 3a shows the schematic of the FE model, including the upper loading platen and the lower supporting platen, with the lattice specimen placed between the two platens. To obtain the constitutive parameters of the base material, three identical dog-bone specimens were fabricated and stretched according to the GB/T 228 standard, using the same SLM processing parameters as for the lattice specimens. The true stress–strain curves obtained from the tensile tests are shown in Figure 3b, indicating satisfactory reproducibility of the printed material’s mechanical properties. The density *ρ* is 7.98 g/cm^3^, the Poisson’s ratio *ν* is 0.3, the Young’s modulus *E* is 60 GPa, and the initial yield strength is 559 MPa. The contacts between the loading and supporting platens and the lattice specimens, as well as between the lattice components and themselves, were modeled using the general contact algorithm, with a friction coefficient of 0.2. In order to balance the accuracy and efficiency of FE simulations, a mesh convergence analysis was conducted. Figure 3c illustrates that the energy absorption and computation time of the BCE^NH2V2^ lattice varied as the mesh size ranged from 0.1 to 0.4 mm. It indicates that the energy absorption converges for mesh sizes below 2 mm. Considering both the computational accuracy and efficiency, a mesh size of 0.2 mm was chosen for the finite element simulations. The element type used is C3D4. The lower supporting platen was fully constrained, while the upper loading platen was moved downward at a velocity of 0.1 mm/s. Although the compression speed is higher than that in the quasi-static compression experiment, the ratio of the kinetic energy to the internal energy during the compression is far less than 5%, as shown in Figure 3d, satisfying the quasi-static condition as demonstrated in the literature. The boundary effect was also investigated. As shown in Figure 3e, the experimental tests were carried out for specimens containing 27, 64, and 125 cells, and the resulting stress–strain curves indicate little difference between the three. Considering the computational cost, a 3 × 3 × 3 lattice was used for all the tests, as well as the FE simulations.

### 2.3. Compression Performance Metrics

To quantitively evaluate the mechanical performance of the lattice structures, in this paper, we refer to various metrics including SEA, specific stiffness (SE), and CFE.

SEA is defined by the following equation:

(2) $$EA=\int_{0}^{x_{d}}F(x)dx$$

(3) $$SEA=\frac{EA}{M}$$

Lattice structures demonstrate strong energy absorption capabilities in load-bearing and impact protection applications. During this process, lattice structures typically undergo a reversible elastic deformation stage followed by a plastic plateau stage. In previous studies, the work of external forces during the plastic stage (i.e., from the end of the elastic stage to the densification point) was usually used for the evaluation of energy absorption of the lattice structures [4]. This evaluation criterion is also adopted in this paper, where *x*_d_ represents the displacement at the densification point. EA denotes the energy absorbed during the compression process, which is obtained by integrating the force *F* with respect to the displacement *x*. SEA represents the energy absorption per unit mass. Due to the high sensitivity of energy absorption of the lattice structures to their densities, SEA is more suitable for assessing the energy absorption capacity of lattices.

SE is used for evaluating the load-bearing capacity of the lattice structures and is an important performance indicator, especially in aerospace applications. A higher SE indicates a lighter weight for the same modulus, or a larger modulus for the same weight. For lattice structures, SE is defined as the ratio of the equivalent modulus *E* to the equivalent density *ρ*, as in the following equation:

(4) $$SE=\frac{E}{\rho }$$

CFE is defined by the following equation:

(5) $$CFE=\frac{PCF}{MCF}$$

where MCF represents the mean crushing force and PCF represents the peak crushing force. CFE is typically used to assess the crushing stability of the specific material or structure under the compression load. A higher CFE indicates a more stable crushing mode.

## 3. Results and Discussion

### 3.1. Comparison between Experiments and FE Simulations

**Figure 4:**
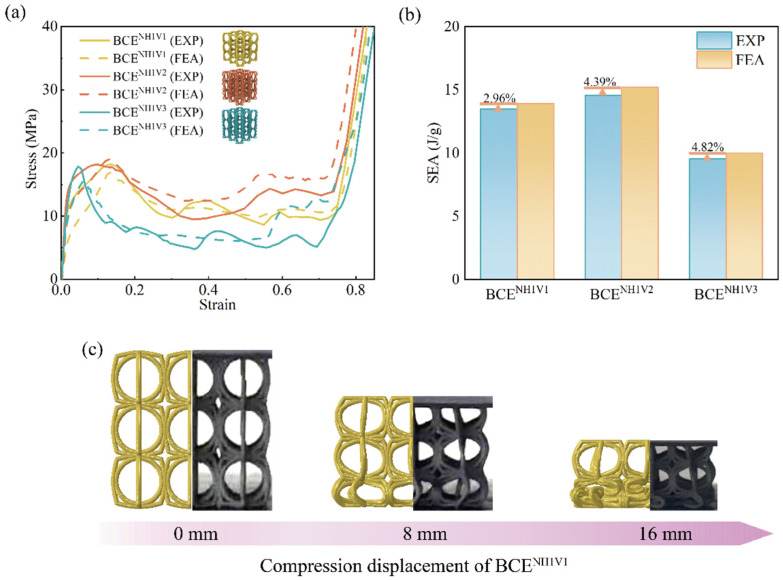
Comparison between the experimental tests and the numerical simulations of the BCE^NH1^ lattices for: (**a**) stress–strain curves; (**b**) SEA; (**c**) deformation modes.

**Figure 5:**
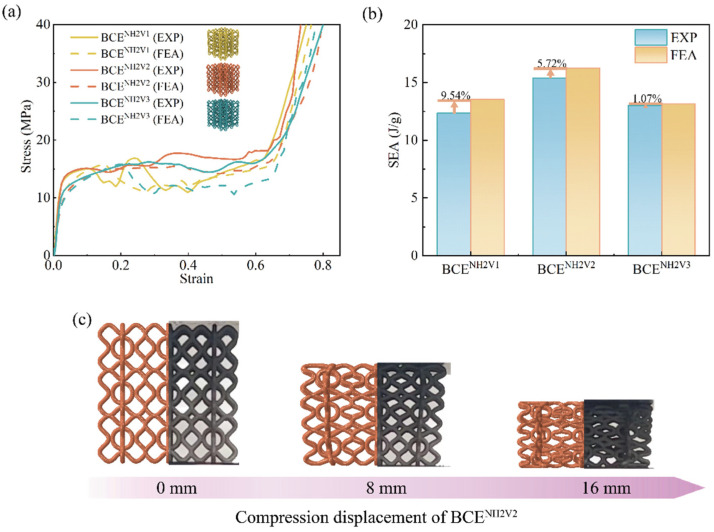
Comparison between the experimental tests and the numerical simulations of the BCE^NH2^ lattices for: (**a**) stress–strain curves; (**b**) SEA; (**c**) deformation modes.

**Figure 6:**
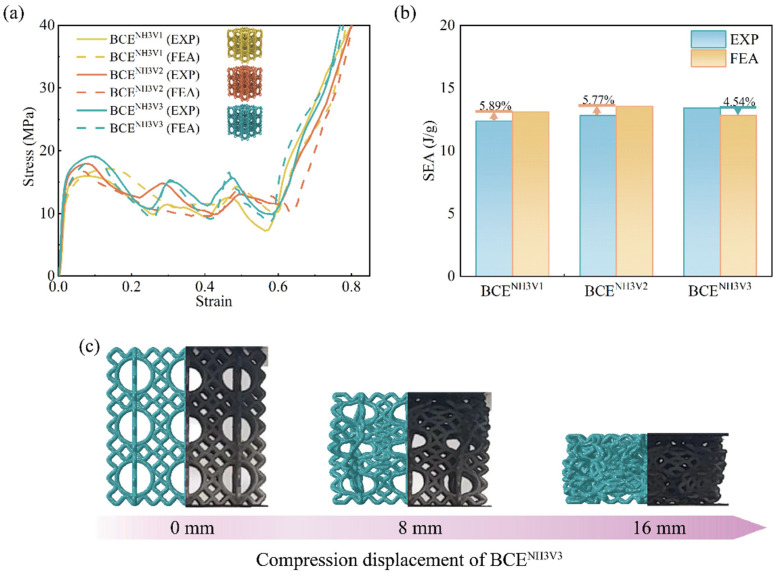
Comparison between the experimental tests and the numerical simulations of the BCE^NH3^ lattices for: (**a**) stress–strain curves; (**b**) SEA; (**c**) deformation modes.

In order to validate the FE numerical model, the obtained stress–strain curves, SEA, and the deformation modes from the FE simulations are compared with those from the experimental tests for BCE lattices with nine different *N* values. The results are shown in Figure 4, Figure 5 and Figure 6 for the specimens with *N_H_* equal to 1, 2, and 3, respectively. From the stress–strain curves shown in Figure 4a, it can be observed that the BCE structures with *N_H_* = 1 all exhibit a stress response with a relatively high initial peak, followed by a sudden drop. On the other hand, the BCE structures with *N_H_* = 2 exhibit no obvious initial stress peaks, but rather a stable stress response without a catastrophic drop in the plateau stage, as shown in Figure 5a, while the BCE structures with *N_H_* = 3 exhibit a stress response of fluctuating character during the plateau stage. These characteristics of stress responses are reflected in both experimental tests and numerical simulations. This indicates that the BCE structures with different *N_H_* values exhibit distinct stress responses and deformation modes, which will be further analyzed in Section 3.3. In addition, from the stress–strain curves in Figure 4, Figure 5 and Figure 6, we can see that the relative deviation of SEA between experiments and FE simulations is less than 10% for various BCE structures, and the compression deformation results also match well. These consistencies demonstrate the accuracy and reliability of the FE numerical simulations.

### 3.2. Comparison between BCE Lattices and Traditional Lattice Structures

**Figure 7:**
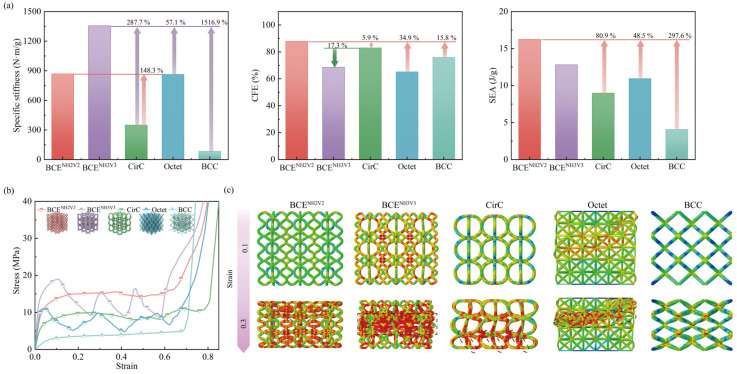
Comparison of BCE with other lattice structures for (**a**) compression performance metrics; (**b**) stress–strain curves; (**c**) deformation modes.

To demonstrate the advantages of the proposed BCE lattice structures, specimens of traditional bending-dominated lattice structures (Circular cell-based (CirC) and BCC) and traditional stretching-dominated lattice structures (Octet) with the same relative density (8%) were established and compared with the BCE specimens, as shown in Figure 7 and Table 2. The Octet lattice is characterized by its unit cell composed of two Platonic configurations, i.e., the tetrahedron and the octahedron. The CirC lattice structure has a circular-based cell topology, with the unit cell consisting of two identical circles, each belonging to a unique plane interconnected perpendicularly (Figure 7b). For this comparison, BCE^NH2V2^ and BCE^NH3V3^ were selected because they, respectively, exhibit prominent bending-dominated and stretching-dominated characteristics. The stress–strain curve of a lattice structure can be roughly divided into three stages, namely (1) the elastic stage, (2) the plateau stage, and (3) the densification stage. The BCE lattice structures, due to their numerous curved components, can utilize more materials to bear the loads during the compression process. Therefore, the stress is more uniformly distributed in the BCE structures, resulting in higher specific stiffness and higher plateau stress. 

The BCE^NH3V3^ structure exhibits a typical stretching-dominated stress response. It is characterized by the rapid increase of stress in the elastic stage, rendering a high stiffness and a high initial peak stress. As shown in Figure 7a, the specific stiffness of BCE^NH3V3^ is improved by 287.7% and 57.1% compared with CirC and Octet, respectively. From the deformation mode shown in Figure 7c, a layer-by-layer progressive collapse is observed, which can explain the evident undulations in the stress–strain curve during the plateau stage. As one layer of the structure is collapsing, an abrupt loss of load-bearing capacity takes place, leading to the sudden stress drop. After the layer is fully collapsed, the load is transferred to the subsequent layer, leading to the increase in stress response. Thus, an undulation-type stress–strain curve is generated. After the collapse of the three layers, the structure enters the densification stage, where a significant increase in stress takes place due to the interactions of the tightly compacted structure components. The above deformation mechanisms result in a high initial yield stress but low CFE for the BCE^NH3V3^ structure, indicating poor deformation stability. Nevertheless, compared to the traditional stretching-dominated structure Octet, BCE^NH3V3^ exhibits superior energy absorption performance. Although its deformation mode is characterized by layer-by-layer localization, the deformation distribution is not as concentrated as in the Octet structure. During the collapse of the BCE structures, components of different levels take part in the load bearing, resulting in a higher overall stress response.

Localized deformation tends to lead to catastrophic failure, whereas delocalized deformation often results in stable stress responses. The bending-dominated BCC lattices, for example, exhibit relatively stable stress responses and obvious stress plateaus. However, their energy absorption is solely due to the plastic hinges formed at the nodes, resulting in low material utilization efficiency. As shown in Figure 7, the BCE^NH2V2^ structure takes on a delocalized deformation mode while maintaining a relatively high stress response. No sudden drop of stress is observed during the compression process. It possesses both of the advantages of high SEA and high CFE compared to traditional structures. The improvement in compression performance is attributed to the interaction between the elliptical and curved beam components of the BCE lattice structure, leading to the formation of more plastic hinges and more uniform stress distribution during the compression, and thus the effective utilization of the material and a stable stress response. 

### 3.3. Parametric Study for BCE Structures

#### 3.3.1. Effect of the Number of Strut Waviness *N*

**Figure 8:**
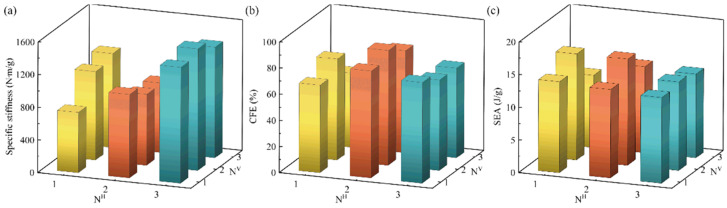
Comparison of BCE structures with different numbers of strut waviness *N* for (**a**) specific stiffness; (**b**) CFE; (**c**) SEA.

To evaluate the effect of the number of strut waviness *N* on the compression performance of BCE lattice structures, the specific stiffness, CFE, and SEA are compared between nine BCE lattices with different *N_H_* or *N_V_* but the same relative density and curve amplitude, as shown in Figure 8 and Table 3. From Figure 8a, it can be seen that when *N_H_* = 1, the specific stiffness increases with the increase in *N_V_*, while for *N_H_* = 2 or 3, the specific stiffness does not show a monotonic relationship with *N_V_*. Compared with *N*_V_, the specific stiffness is more sensitive to *N*_H_ and is at its maximum at *N_H_* = 3. Generally, curved struts are more prone to bending deformation due to their initial curvature, which tends to decrease the structural stiffness. However, as *N*_H_ increases, the angle between the major parts of the curved struts and the compression direction decreases, approaching the situation of straight vertical rods. This can explain why the structure exhibits higher specific stiffness at *N_H_* = 3 compared with *N_H_* = 1 or 2.

**Figure 9:**
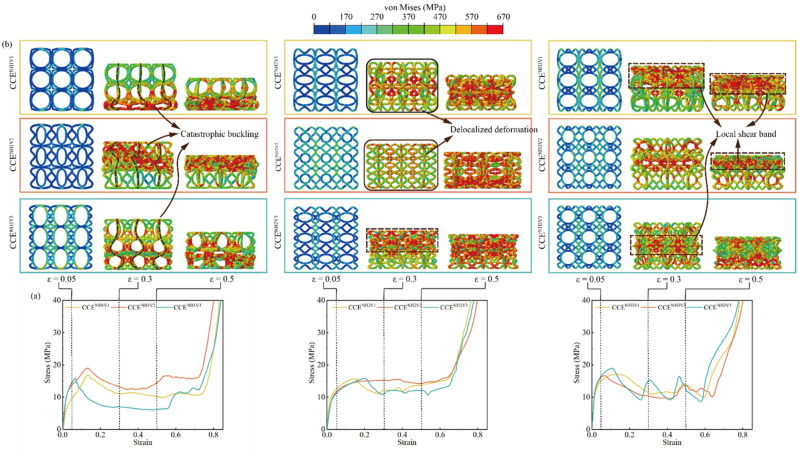
Effect of the number of strut waviness *N* on (**a**) the deformation modes; (**b**) the stress–strain curves.

From Figure 8b, it can be seen that the BCE lattice structures with *N_H_* = 2 exhibit higher CFE compared with the cases of *N_H_* = 1 or 3. The CFE metric is largely determined by the local deformation modes of the lattice structure. As shown in Figure 9, the number of waviness *N* has a strong influence on the deformation mode of the structure. From Figure 9a, the structures with *N*_H_ = 1 all exhibit evident local buckling (indicated by the dashed lines) during the compression process, leading to a significant stress drop after reaching the initial peak stress. The deformation mode of local buckling is also evident for the structures with *N_H_* = 3, as shown in the deformation concentration bands indicated by the dashed square frames in Figure 9b. However, for the structures with *N_H_* = 2 (BCE^NH2V1^ and BCE^NH2V2^), a delocalized deformation mode is observed (indicated by the solid square frames), with relatively uniformly distributed stress and strain. The buckling and the resulting deformation localization for BCEs with *N_H_* = 1 or 3 can be attributed to the vertically oriented major axis of the central ellipses, making the structures more similar to the stretching-dominated lattices. Typically, stretching-dominated lattice structures exhibit conspicuous deformation localization, leading to layer-by-layer collapse and significant fluctuations in stress response, which renders relatively low CFE values. In addition, large cavities are formed by the large ellipse beams in some BCE^NH1^ and BCE^NH3^ structures, further concentrating the material distribution and deteriorating the deformation localization. On the other hand, for the BCE structures with *N_H_* = 2, the ellipses are oriented with the major axes along the horizontal direction (BCE^NH2V1^ and BCE^NH2V3^), or the central cavities formed by the ellipse beams are sufficiently small (BCE^NH2V2^), making the lattice structures more similar to the bending-dominated cases. A uniformly distributed deformation is observed in BCE^NH2^, indicating more efficient material utilization and leading to a higher CFE compared to BCE^NH1^ and BCE^NH3^. Unlike the traditional bending-dominated lattice structures such as Kelvin and BCC, which exhibit stable stress responses but relatively low strength and energy absorption capacities, the BCE^NH2V1^ and BCE^NH2V2^ structures possess higher CFE values, along with higher SEA, due to their delocalized deformation modes. This further demonstrates the advantages of BCE structures.

#### 3.3.2. Effect of the Amplitude of the Strut Waviness *A*

To investigate the effect of the strut waviness amplitude *A* on the compression performance of the BCE lattice structures, the structure BCE^NH2V2^ is taken as the study objective with both *N_H_* and *N_V_* kept as 2, and *A*_H_ and *A*_V_ varying from 0.5 to 2.0 mm. Next, we introduce the dimensionless parameters ${\bar{A}}_{H}$ and ${\bar{A}}_{V}$, as in the following equation:

(6) $${\bar{A}}_{H}=\frac{A_{H}}{L_{H}}$$

(7) $${\bar{A}}_{V}=\frac{A_{V}}{L_{V}}$$

In the above definitions, *L*_H_ and *L*_V_ are the horizontal and vertical lengths of the BCE lattice unit cell, which are both 10 mm. In this work, ${\bar{A}}_{H}$ and ${\bar{A}}_{V}$ vary from 0.05 to 0.2.

**Figure 10:**
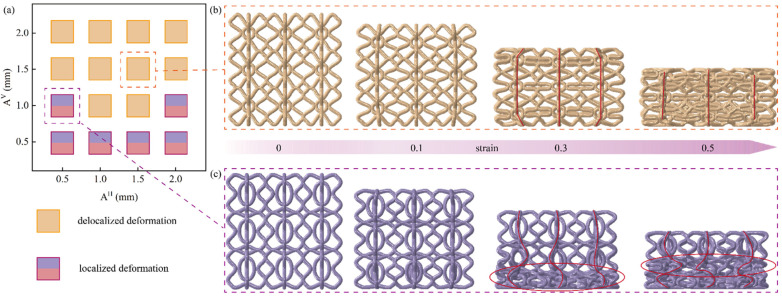
Effects of ${\bar{A}}_{H}$ and ${\bar{A}}_{V}$ on the compression performance indicators of BCE^NH2V2^: (**a**) classification chart of deformation modes; (**b**) deformation modes for ${\bar{A}}_{H}$ = 0.15 and ${\bar{A}}_{V}$ = 0.15; (**c**) deformation modes for ${\bar{A}}_{H}$ = 0.05 and ${\bar{A}}_{V}$ = 0.1.

Figure 10a shows the classification of the deformation modes of BCE structures with different ${\bar{A}}_{H}$ and ${\bar{A}}_{V}$. The results indicate that the amplitude of waviness along the vertical struts ${\bar{A}}_{V}$ has a significant influence on the deformation mode of the BCE^NH2V2^ lattice structure. For ${\bar{A}}_{V}$ larger than 0.1, a delocalized deformation mode is observed. For example, when ${\bar{A}}_{H}$ = ${\bar{A}}_{V}$ = 0.15 mm, the BCE lattice structure is deformed uniformly without catastrophic layer-by-layer collapse, as shown in Figure 10b. From the marker of the solid red line in Figure 10b, it is noteworthy that the vertical material plane is largely kept straight during the compression, indicating that no buckling occurred. On the other hand, for ${\bar{A}}_{V}$ smaller than 0.1 mm, the BCE^NH2V2^ lattice structure exhibits localized deformation during compression. As an example of the critical situation, the BCE^NH2V2^ lattice structure with ${\bar{A}}_{H}$ = 0.05 and ${\bar{A}}_{V}$ = 0.1 is taken to demonstrate the evolution of deformation during the compression process, as shown in Figure 10c. When the strain reaches 0.3, catastrophic collapse occurs in the bottom layer of the lattice first (as indicated by the red elliptical circle in Figure 10c). At this point, the second layer begins to collapse, characterized by the evident bending of the original straight solid red line indicating a vertical plane of the material. When the strain reaches 0.5, the second layer of the structure is fully compacted, as indicated by the red ellipse circle, and the collapse continues to take place in the top layer. The distinct deformation modes exhibited by the BCE structures with small and large strut waviness amplitudes can be explained by the different propensity of buckling deformation occurring. A small ${\bar{A}}_{H}$ corresponds to a small curvature of the vertical wavy strut, making the lattice structure more similar to straight simply cubic, and buckling deformation is more prone to occurring towards one side under the compression load. On the contrary, when ${\bar{A}}_{V}$ is large, the smooth bending of the significantly curved lattice struts effectively precludes the occurrence of buckling deformation during compression.

**Figure 11:**
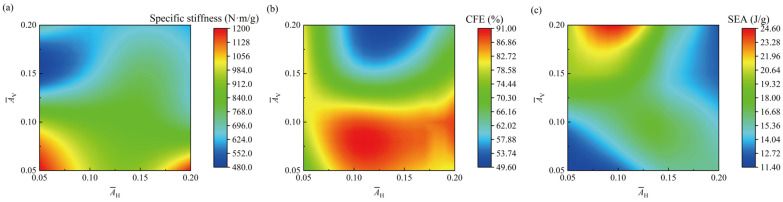
Influence of ${\bar{A}}_{H}$ and ${\bar{A}}_{V}$ on the compressive performance indicators: (**a**) specific stiffness; (**b**) CFE; (**c**) SEA.

Figure 11a–c shows the contour charts of the performance indicators of the BCE^NH2V2^ lattice structure on the plane of ${\bar{A}}_{H}$ and ${\bar{A}}_{V}$, for specific stiffness, CFE, and SEA, respectively. From Figure 11a, it can be seen that the specific stiffness of the BCE^NH2V2^ lattice structure decreases monotonically with the increase of ${\bar{A}}_{V}$. As illustrated by Figure 10, for small ${\bar{A}}_{V}$, the deformation mode of the BCE^NH2V2^ lattice structure is more close to the stretching-dominated type, resulting in higher stiffness. When ${\bar{A}}_{V}$ decreases from 0.2 to 0.05, the specific stiffness increases by 72.4%. From Figure 11b, it can be seen that the variation of CFE is more complex. For the cases of ${\bar{A}}_{V}$ = 0.1 and ${\bar{A}}_{H}$ = 0.1–0.15, the BCE^NH2V2^ lattice structure exhibits exceptionally high CFE of over 90%. However, as ${\bar{A}}_{V}$ increases from 0.1 to 0.2, the CFE indicator decreases by 45.5%. This indicates that an increase in ${\bar{A}}_{V}$ does not always benefit the improvement of the CFE of the structure. The selection of ${\bar{A}}_{H}$ = ${\bar{A}}_{V}$ = 0.1 is recommended for the BCE^NH2V2^ structure to achieve a stable stress response and a stable deformation mode. The energy absorption capacity at different ${\bar{A}}_{H}$ and ${\bar{A}}_{V}$ is shown in Figure 11c. It can be seen that the variation tendency of SEA with ${\bar{A}}_{H}$ or ${\bar{A}}_{V}$ is not monotonic. For instance, when ${\bar{A}}_{H}$ = ${\bar{A}}_{V}$ = 0.05, SEA is only 11.4 J/g; when ${\bar{A}}_{H}$ and ${\bar{A}}_{V}$ increase to 0.15 mm, SEA increases to 19.3 J/g; but when ${\bar{A}}_{H}$ and ${\bar{A}}_{V}$ further increase to 0.2, SEA is reduced back to 12.2 J/g. This is because when the strut waviness amplitude *A* is too small or too large, the corresponding ellipses are too large or too small compared to the curved beam region, which is not conducive to utilizing the interaction between the curved beams and the ellipses, thereby compromising energy absorption. Generally, when ${\bar{A}}_{V}$ is small, SEA shows an increasing trend with increasing ${\bar{A}}_{H}$, whereas when ${\bar{A}}_{V}$ is large, SEA shows a decreasing trend with increasing ${\bar{A}}_{H}$. The maximum value of SEA takes place at ${\bar{A}}_{H}$ = 0.1 and ${\bar{A}}_{V}$ = 0.2, reaching 24.6 J/g. However, under these parameters, the specific stiffness is only 635.6 N·m/g. Conversely, at the parameters where the specific stiffness is maximized (${\bar{A}}_{H}$ = ${\bar{A}}_{V}$ = 0.05), specific energy absorption is minimal, only 11.4 J/g. Therefore, a moderate value of ${\bar{A}}_{H}$ and ${\bar{A}}_{V}$ (within the range of 0.1 to 0.15) is more beneficial for achieving both high specific stiffness and specific energy absorption.

In summary, for the BCE^NH2V2^ lattice structure, ${\bar{A}}_{V}$ plays a significant role in adjusting its compression performance and deformation mode. To ensure that the BCE^NH2V2^ lattice structure possesses both high energy absorption and high specific stiffness while maintaining a stable deformation mode, ${\bar{A}}_{H}$ and ${\bar{A}}_{V}$ should be between 0.1 to 0.15.

## 4. Conclusions

In this paper, we propose a bio-inspired curved-elliptical lattice structure to enhance the mechanical performance and deformation stability of three-dimensional lattice structures. The following conclusions can be drawn:

(1) The proposed BCE structures with different geometric parameters exhibit different deformation modes. By adjusting the number and amplitude of the strut waviness, a stable delocalized deformation mode can be achieved, avoiding catastrophic collapse during compression. The CFE of BCE is higher than that of Octet, CirC, and BCC, with improvements of 34.9%, 5.9%, and 15.8%, respectively.
(2) The proposed BCE structures exhibit high compression performance in terms of SEA and specific stiffness. The SEA of BCE is higher compared to Octet, CirC, and BCC, with increases of 48.5%, 80.9%, and 297.6%, respectively. Similarly, the specific stiffness is improved by 57.1%, 287.7%, and 1516.9%, respectively. Notably, the BCE structure can achieve simultaneous enhancements of SEA and specific stiffness at appropriate geometric parameters.
(3) The number *N* and the amplitude *A* of the strut waviness are important parameters affecting the compression performance of BCE structures. From the parametric analysis, the deformation mode of BCE structures is most sensitive to the waviness amplitude of the vertical struts and the waviness number of the horizontal struts.

In summary, the proposed BCE lattice structures demonstrate excellent compression performance and controllable deformation modes, providing new pathways for designing high-performance lattice structures.

## Figures and Tables

**Table 1:** Basic parameters of laser selective melting forming equipment.

Technical Specifications	SLM
Build envelope (*L* × *W* × *H*)	280 × 280 × 365
Variable layer thickness/μm	20–90
Real build rate/cm^3^·h^−1^	Up to 113
Beam focus diameter/μm	80–115
Average inert gas consumption in process/L·min^−1^	13(Argon)
Compressed air requirement/bar	ISO 8573-1:2010 [1:4:1] 7
Machine dimensions (*L* × *W* × *H*)/mm	4150 × 1200 × 2525

**Table 2:** Comparison of BCE with other lattice structures for specific stiffness, CFE, and SEA.

	Specific Stiffness (N∙m/g)	CFE (%)	SEA (J/g)
BCE^NH2V2^	868.8	87.9	16.3
BCE^NH3V3^	1356.6	68.6	12.8
CirC	349.9	82.9	8.9
Octet	863.8	65.2	10.9
BCC	83.9	75.9	4.1

**Table 3:** Comparison of BCE structures with different numbers of strut waviness *N* for specific stiffness, CFE, and SEA.

	Specific Stiffness (N∙m/g)	CFE (%)	SEA (J/g)
BCE^NH1V1^	741.4	67.1	13.9
BCE^NH1V2^	1082.9	77.3	16.3
BCE^NH1V3^	1149.9	56.6	11.0
BCE^NH2V1^	1021.9	81.8	13.5
BCE^NH2V2^	868.8	87.9	16.3
BCE^NH2V3^	848.2	78.0	13.2
BCE^NH3V1^	1418.8	77.1	13.1
BCE^NH3V2^	1491.8	69.2	13.6
BCE^NH3V3^	1356.6	68.6	12.8

## Data Availability

Dataset available on request from the authors.
